# Supplementary material for: Delta-catenin is required for cell proliferation in virus-positive Merkel cell carcinoma cell lines but not in human fibroblasts
Source: mBio. 2025 May 23;16(6):e00832-25. doi: 10.1128/mbio.00832-25 (PMC12153310; doi:10.1128/mbio.00832-25)
Supplement: Supplemental material — Supplemental figures and tables. [file mbio.00832-25-s0001.pdf]

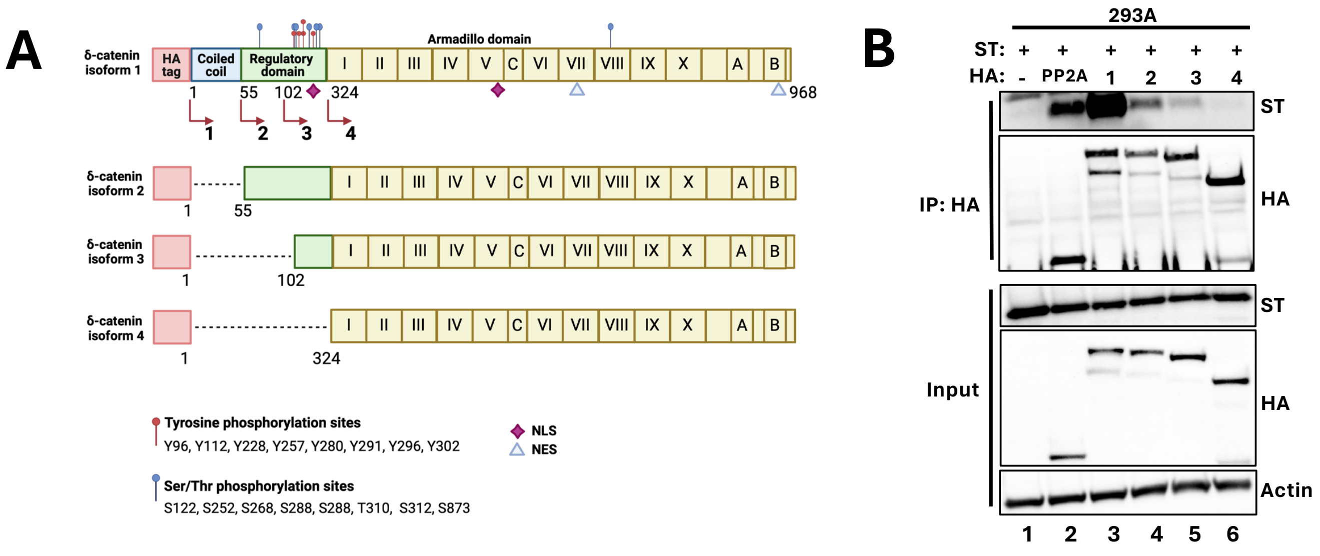

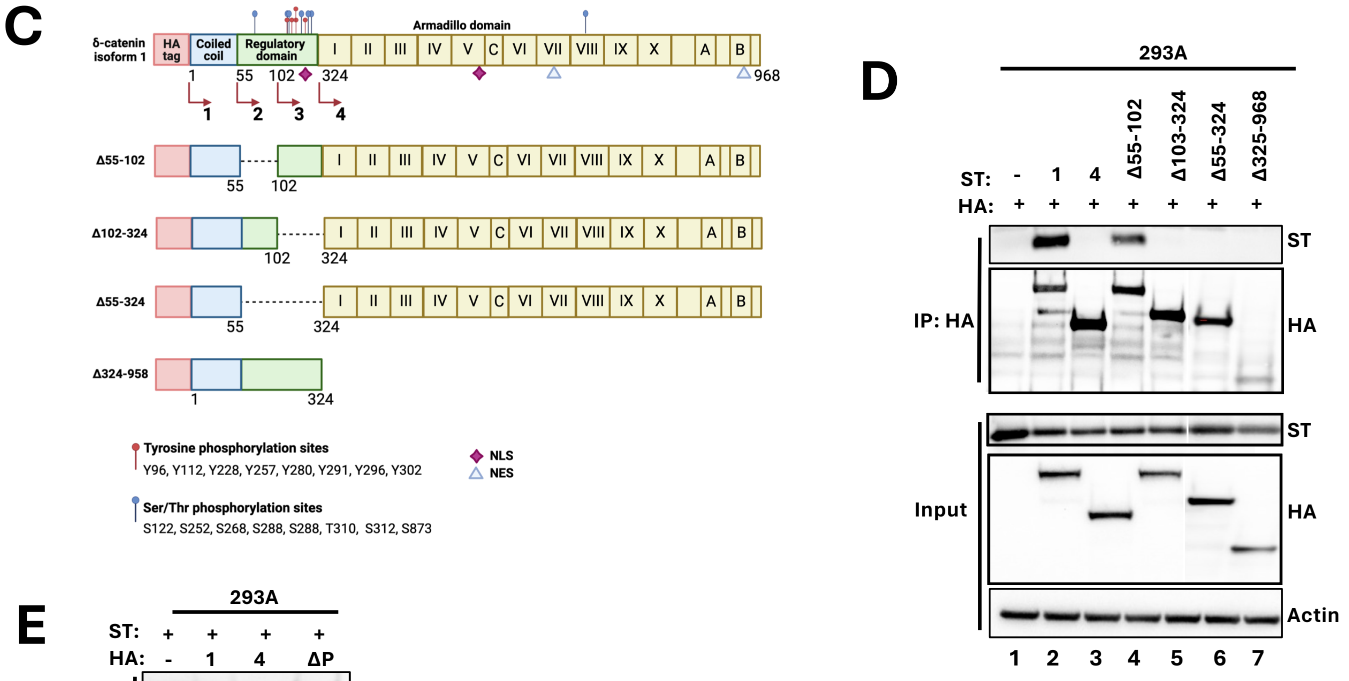

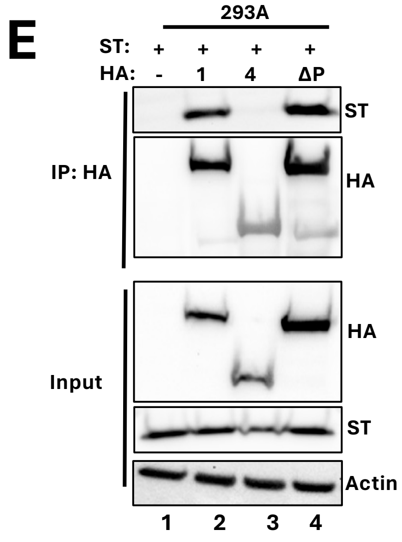

Figure S1: δ-catenin isoform 1 binds strongly to MCPyV ST. A) Schematic representation of δ-catenin isoforms. ATG 1−4 represent the positions of the four alternatively used start codons. **B)** Western blots of HEK293A cell lysates transfected with either ST (2ug) or HA- δ-catenin isoform 1-4 (5ug) or HA-PP2AC (5ug). Lysates were immunoprecipitated with HA antibody and blotted for MCPyV ST. **C)** Schematic representation of δ-catenin N-terminal deletions. **D)** Western blots of HEK293A cell lysates transfected with ST (2ug) and HA- δ-catenin mutants (5ug). Lysates were immunoprecipitated with HA antibody and blotted for MCPyV ST. **E)** Western blots of 293A cell lysates transfected with ST (2ug) and HA- phospho-mutant of δ-catenin isoform 1 (ΔP) (5ug). Tyrosine phosphorylation sites were mutated to phenylalanine, and serine/threonine sites were mutated to alanine. Lysates were immunoprecipitated with HA antibody and blotted for MCPyV ST.

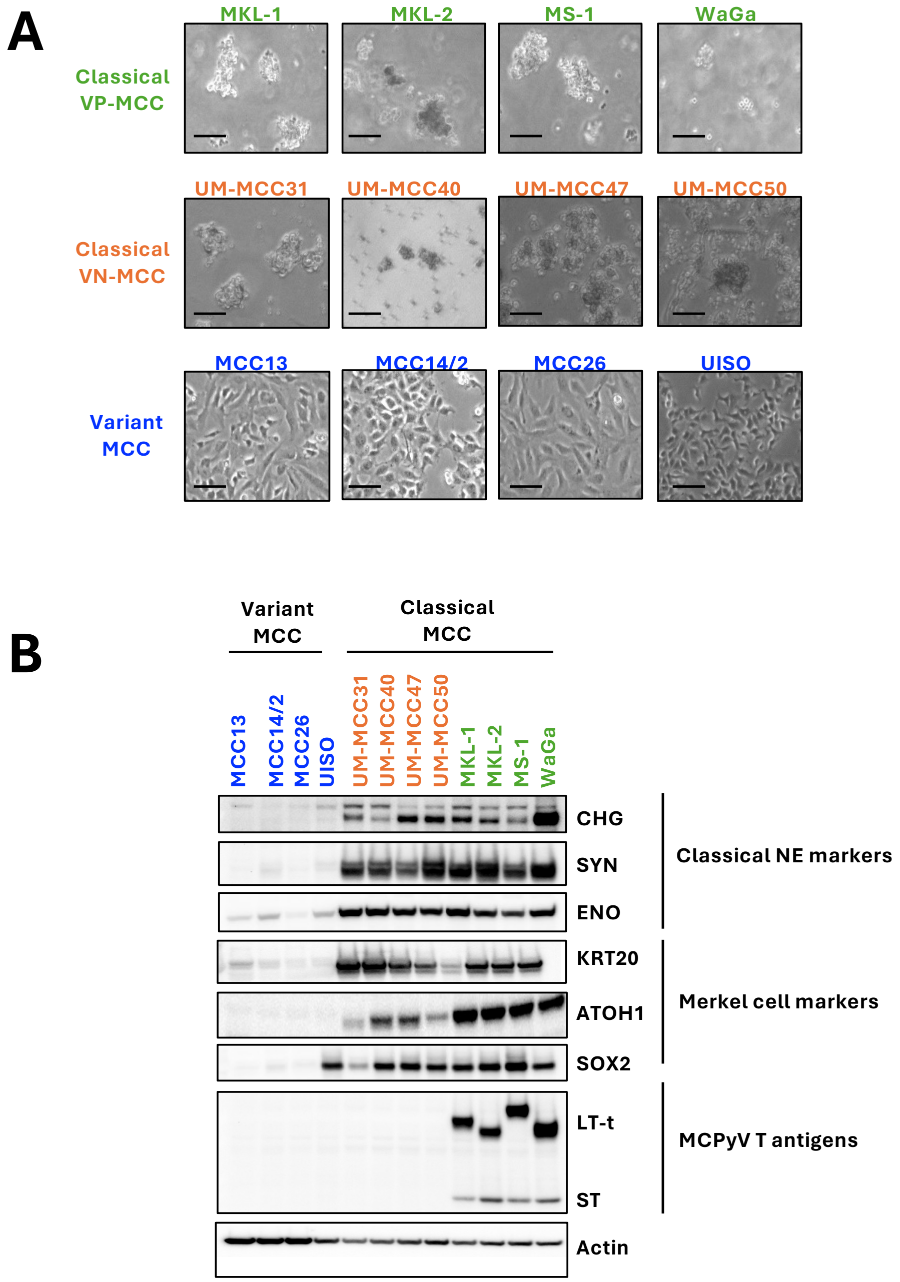

Figure S2: Merkel cell carcinoma (MCC) cell lines, including variant, classical virus-negative (VN) and classical virus-positive (VP). A) Morphologic comparison of the 12 cell lines. **B)** Expression of classical neuroendocrine (NE) markers, Merkel cell markers and MCPyV Small T (ST) and truncated large T (t-LT) antigens across the panel of 12 cell lines. CHG: Chromogranin A . SYN: Synaptophysin. ENO: Enolase 2. KRT20: Keratin 20**.**

Human Cyclin B3 promoter

CTCCCTACTCCAGACAACTAGGACATCCCTGAATTCTGTATGTTCATGC
CTCTGCCTGGGCTCGGCTTTCTTTGCCTGGAAACCTCCCAGTCAACT
TGGGAACCTGTTTGTCATTTTAAGTCCTGATCACATGTTACTTCCTTTG
TGAAGCCTTCCTCCAGCCCCTGTCCCCAACAAGATTACTGGCTTTCCA
TTATGTTTCTTTTTTCTTTTCTTTTTTTTTTTTAGTTTGGCATGACAGACA
CATATAAATTTTATTATGATTTAATCATTATTTCTATCCTTTTCCTTGAACA
AAGTCTATAAAAGAATTTTAGTTGTATTTTTCCAGGTAGTTGGATTTTTT
AAAAAACATTTTGCCTCTATCAAAATACTTCAGGAATACATTTTTAGCAG
TCATATTAAGCATCACAGACACATTATCAACCTTTACTTAGATTTAGCCA
TATATTTTATTGGTTTCTTTGCTCAACTGCTTACTTTTCAACTGTTTTCTC
ATTTTTCTGTTTCTCCTCCATCTTCCTTTCTCTTCCCCCTACTCCTTCAC
CATCTTCGTCCTTAGGTAATTTTAGTTTGCCCTCACATCTGCATAATTGG
TAAAGTATAGAATTCTAGGTGAAAAATCTTTTTCCCTAGAAACTATGTAA
ACGGTGTTTCACTGCCTTCTGTCATTTGGTGTGAAGTTACTAATCATTA
TGTTTCTATAGCACTTAGTTTGCGCTGCTATAAAGTGCCATGGACTGGG
TGGCATTTAGTTCTCACAGTTCTGGAGGCTGGGAAATCCAAGATCAAG
TTCTTATGTCAAATGATTTTTTAATAAGTTAGTTTATTTTGC

Human Cyclin A2 promoter

ACAATTTCTGGTTACTATGAATAAACGCCTAAATGTTAAGATGACATTA
CAGTCTTGACACTTGAGTACTGTATTACTATGTGAGCTCCGTGTTAAA
TAATTTATGCACATTATTTAATCCTAACAACCATATGACTGTAGTTATT
AGTCCCTATTAACACATAAGAAAACGGAGAATCGGAGATACTGAAAAA
CGTGCCCCAGATTTTAGACCTTTGGAAAAAGTCACTTAAGCTAACTAG
ACGTCCCAGAGCTAAAGGCTGGGCAACCCAAATGATAGTCGCCAAAG
TTTAATTCCGTTTAATTCCCTAAAAGGCTTAGAGTCAGCCTTCGGACA
GCCTCGCTCACTAGGTGGCTCAGCTTAAAATAATCGGAAGCGTCGGG
CCCTAAATCCTACCTCTCCCCGCCCCGCGCAGGCGTTTTCTCCCGCC
CCAGCCAGTTTGTTTCTCCCTCCTGCCCCGCCCCTGCTCAGTTTCCTT
TGGTTTACCCTTCACTCGCCTGCGACCCTGTCGCCTTGAATGACGTC
AAGGCCGCGAGCGCTTTCATTGGTCCATTTCAATAGTCGCGGGATAC
TTGAACTGCAAGAACAGCCGCCGCTCCGGCGGGCTGCTCGCTGCAT
CTCTGGGCGTCTTTGGCTCGCCACGCTGGGCAGTGCCTGCCTGCGC
CTTTCGCAACCTCCTCGGCCCTGCGTGGTCTCGAGCTGGGTGAGCG
AGCGGGCGGGCTGGTAGGCTGGCCTGGGCTGCGACCGGCGGCTAC
GACTATTCTTTGGCCGGGTCGGTGCGAGTGGTCGGCTGGGCAGAGT
GCACGCTGCTTGGCGCCGCAGGCTGATCCCGCCGTCCACTCCCGGG
AGCAGTG

Human CDC6 promoter

ACTGGTGATAAAGACACCCCGCGTGCCTGGAGGGAGGAAACTAGAAGTTCTAT
ATAAATCAATTCATGTAACTTTTTTTTTTTTTTTGAGACGGAGTCTCGCTCTTGTCG
CCCAGGCTGTAGTGCAGTGGTGCGATCTCGGCTCACTGCAACCTCTGCCTGCC
GGGTTCAAGCCATTCTCCTGCCTCAGCCTCTTCATGTAGCTGGGACTACAGGC
GCCCGCCACCACGTCCGGCTAATTTTTTTTTTTTTTTTTGAGACGGAGTCTCGCT
CGGTCTCCCAGGCTGGAGTGCAGTGGCGCGATCTCGGCTCACTGCAAGCTCC
GCCTCCCGGGTTCATGCCATTCTCCTGCCTCAGCCTCCCGAGTAGCTGGGACT
ACAGGCGCCCACCACCACGCCCGGCTAATTTTTTGCATTTTTAGTAGAGACGGG
GTTTCACCGTGTTAGCCAGGATGGTCTCGGCCCGTCTCGGCCTCCCAAAGTGT
TGGGATTACAGGCGTGAGCCACCGCGTTTGGCCGTAATTTTTGTATTTTTAGTAG
AGACGGGGTTTCTCCATGTTGGTCAGGCTGGTCTCGAACTCCTGACCTCAGGT
GATCCGCCCGCCTCAGCCTCCCAAAGTGCTGGGATTACAGGCGTGGGCCACC
GCGCCCGGCCCATTTCAGGTAACTTGTTATTGCATCGCTCAATTGGTTAAGAACT
TGGGCTCTGGCACCAGATGCCCGGGGTTACATTCCTGTTTTTCTGTGACTTTGG
GCCACTGTTTATCTCAGTTTTCTCATGTGTAAAATAAAGGCTAATAATAGTACCTA
CTCCTAAGATCGTTGTTAAACGAGTTGGTACCTAAAGTGTTTATAACAGCGCCCT
TCCACGTAGTAGGTAGACAACAAATGTCAGCTGTTCTTGCATAGATTTGGATGTG
AAGCAAAAGTGAATGCTAAAATAAGTGTGATAACATAATTAGGAAATAAGTGTTAA
TCATCAGTATAAATAAATTCCATGTTAGTTATTTTCTCTTCACTTTCAC

Figure S3: Nucleotide sequence of the human cyclin B3, cyclin A2 and CDC6 promoter region. Consensus Kaiso binding sites are highlighted in yellow. Arrow indicates the transcription initiation site. Appendix, Table S3.

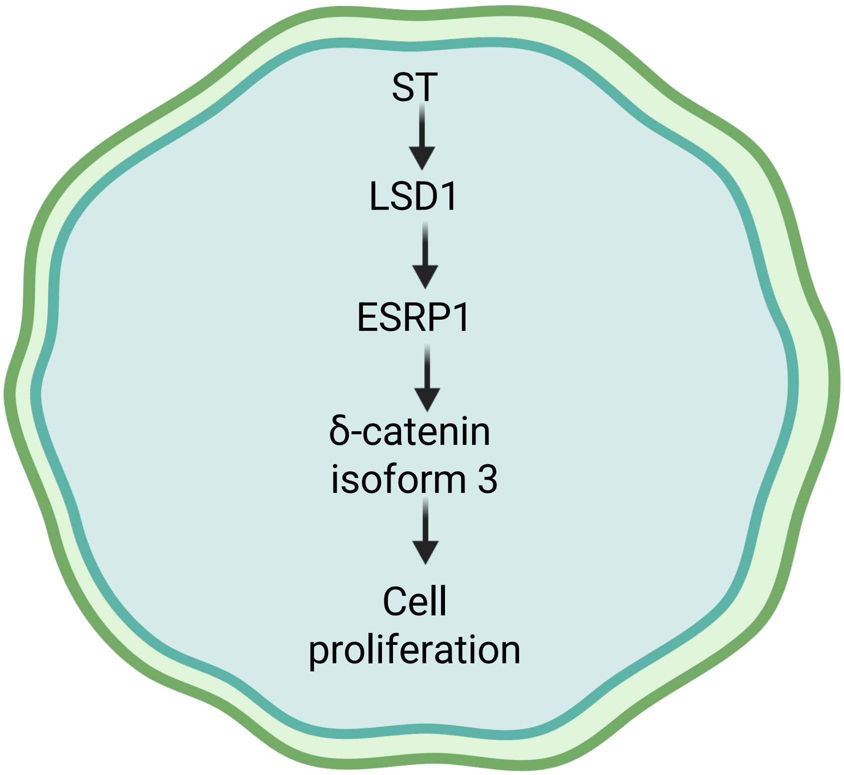

Figure S4: LSD1 regulates the expression of δ-catenin isoform 3 by regulating the expression of ESPR1 in VP-MCC cells.

Table S1. shRNA lentiviral vectors

Target	Catalog number
shRNA non-targeting control	Horizon Discovery #VSC6570
shRNA against δ-catenin	Horizon Discovery #V3SH11252-228652084<br>Horizon Discovery #V3SH11252-229424680
shRNA non-targeting control	Sigma # SHC016
shRNA against ESRP1	Sigma #TRCN0000240875<br>Sigma #TRCN0000245378
shRNA against LSD1	Sigma #TRCN0000046071<br>Sigma #TRCN0000046072

Table S2. Antibodies

Antibody	Company, Catalog number
δ-catenin	Cell signaling #59854
PP2A	Cell signaling #2038
LSD1	Cell signaling #2184
PROM1	Cell signaling #5860
Enolase 2	Cell signaling #24330
Keratin 20	Cell signaling #D9Z1Z
Synaptophysin	Cell signaling #36406
Chromogranin A	Cell signaling #85798
SOX2	Cell signaling #23064
actin-HRP	Cell signaling #5125
Histone 3	Cell signaling #4499
GADPH	Cell signaling #5174
V5-tag	Cell signaling #D3H8Q
HA	BioLegend #16B12
Kaiso	Thermo Fisher #PA5-114790
ESRP1	Thermo Fisher #PA5-25833
ATOH1	Proteintech #21215-1-AP

Table S3. NCBI accession numbers for cell cycle genes

Gene	Ensembl accession numbers for cell cycle genes
CCNB1	ENSG00000134057
CCNB3	ENSG00000147082
CCNA2	ENSG00000145386
CDC6	ENSG00000094804

Table S4. qRT-PCR and PCR primers.

Target	Nucleotide sequence (5' –>3')
δ-catenin isoform 1	F: GGTGGCTGGGATGCTTCTT<br>R: CATGAAGGTAAGGGGCGGAG
δ-catenin isoform 3	F: ACTCCACATTTGAGAACGGC<br>R: AGACATGGCTCCCTCAGGAT
36B4	F: TGCCAGTGTCTGTCTGCAGA<br>R: ACAAAGGCAGATGGATCAGC.
